# The continuing saga of patents and non‐invasive prenatal testing

**DOI:** 10.1002/pd.5450

**Published:** 2019-04-26

**Authors:** Naomi Hawkins, Dianne Nicol, Subhashini Chandrasekharan, Robert Cook‐Deegan

**Affiliations:** ^1^ Law School University of Exeter Exeter UK; ^2^ Law School University of Tasmania Hobart Australia; ^3^ Ripple Effect Communications Inc Rockville Maryland; ^4^ School for the Future of Innovation in Society and Consortium for Science Policy and Outcomes, Arizona State University Tempe Arizona

## Abstract

**Objective:**

This paper examines the Intellectual Property (IP) landscape for non‐invasive prenatal testing (NIPT) in three key regions: the United States; Europe, with particular focus on the United Kingdom; and Australia.

**Method:**

We explore the patent law issues against the commercial and healthcare environment in these regions and consider the implications for development and implementation of NIPT.

**Results:**

There are many patents held by many parties internationally, with litigation over these patents ongoing in many countries. Importantly, there are significant international differences in patent law, with patents invalidated in the USA that remain valid in Europe. Despite the many patents and ongoing litigation, there are multiple providers of testing internationally, and patents do not appear to be preventing patient access to testing for those who can pay out of pocket.

**Conclusion:**

The patent situation in NIPT remains in a state of flux, with uncertainty about how patent rights will be conferred in different jurisdictions, and how patents might affect clinical access. However, patents are unlikely to result in a monopoly for a single provider, with several providers and testing technologies, including both public and private sector entities, likely to remain engaged in delivery of NIPT. However, the effects on access in public healthcare systems are more complex and need to be monitored.

## Abstract 2

What's already known about this topic?

- Non‐invasive prenatal testing (NIPT) is a rapidly growing field of testing, with significant commercial involvement and a number of relevant patents.
- The patents in this field have caused concern about implications for access to testing.
- Recent court decisions and company acquisitions have settled some of these patent disputes and abated some of the concerns about access to testing in the United States, but significant controversy continues in the rest of the world.

What does this study add?

- This study sets out the current law, including recent cases relevant to NIPT, and considers the implications of this law against the commercial environment in the United States, Europe, and Australia, for the future development and delivery of NIPT internationally.

**What's already known about this topic?**

- Non‐invasive prenatal testing (NIPT) is a rapidly growing field of testing, with significant commercial involvement and a number of relevant patents.
- The patents in this field have caused concern about implications for access to testing.
- Recent court decisions and company acquisitions have settled some of these patent disputes and abated some of the concerns about access to testing in the United States, but significant controversy continues in the rest of the world.

**What does this study add?**

- This study sets out the current law, including recent cases relevant to NIPT, and considers the implications of this law against the commercial environment in the United States, Europe, and Australia, for the future development and delivery of NIPT internationally.

## 1 INTRODUCTION

Non‐invasive prenatal genetic testing (NIPT) based on cell free DNA sequencing technology has developed rapidly, and is being incorporated into prenatal screening globally. Intellectual property rights, notably patents, have had, and continue to have, an important influence.^1^, ^2^ Key commercial players hold patent portfolios, and most have engaged in extensive litigation and licensing.^3^, ^4^ Patents have played a key role in shaping the development and delivery of NIPT, and will continue to do so, but that role is neither simple nor certain. This paper examines the IP landscape for NIPT in three key regions: the United States; Europe, with particular focus on the United Kingdom; and Australia. We explore the patent law issues in the context of commercial and healthcare systems in these regions and consider the implications for development and implementation of NIPT. We focus on these jurisdictions because, although they do not represent all countries where NIPT is offered, they represent a cross section of the different legal approaches, different patent office practices and application of patent criteria, key markets for commercial providers, and also the different types of healthcare systems in which NIPT currently operates.^5^

## 2 KEY PATENTS, PRODUCTS, AND PLAYERS

One patent family has been the subject of particular attention in the literature.^6^ An application for US Patent 6 258 540 was filed in 1997. It gave rise to a family of patents in the United States, Australia, and various European countries in broadly similar terms. We use the term “540” to describe this patent family. The invention claimed in 540 arose from research carried out by Dennis Lo and colleagues. The patent was originally owned by Oxford University Innovation, exclusively licensed (except for use in Hong Kong and China), and assigned to Sequenom.^7^ This is the broadest NIPT patent, claiming methods of using cell‐free fetal DNA (cffDNA). If valid, it arguably covers virtually every method of performing NIPT using cffDNA analysis.

Sequenom began offering NIPT in the United States under the brand name MaterniT21 in 2011.The other major entities involved in early NIPT development and delivery in the United States were Verinata Health (Verifi), Ariosa Diagnostics (Harmony), and Natera (Panorama).^1^ In an illustration of the perceived value of NIPT and industry consolidation, Sequenom was acquired by LabCorp, Verinata by Illumina, and Ariosa by Roche. These and other entities involved in NIPT development and delivery have patents, or pending applications, which cover narrower aspects of NIPT, for example, certain types of sequencing techniques or algorithms.

In December 2014, Sequenom and Illumina—arguably the two most prominent companies involved in the development and delivery of NIPT globally—settled their patent disputes by cross‐licensing their patents, including 540.^8^ Through the agreement, Illumina obtained worldwide rights to use the pooled patents for kit tests for NIPT and to license third‐party laboratories to develop and deliver their own laboratory‐developed NIPT. Sequenom and Illumina also retained the right to develop and deliver their own laboratory‐developed NIPT.^9^

There are growing numbers of public and private providers of NIPT globally.^10^ Ariosa (Roche) and Natera remain the major competitors of Sequenom and Illumina in the United States. Premaitha (now Yourgene Health) is a significant late UK entrant into the NIPT market, with its IONA test, offered in Europe and various other countries, but not the United States. BGI Health (Nifty) and Berry Genomics (BambniTest), based in China, are also important players in the NIPT market outside of the United States. Genesis Genetics also offers NIPT in the United Kingdom, based on Verifi technology (Serenity®). In Australia, the Victorian Clinical Genetics Services offers NIPT using the Percept test, based on the original Lo technology, whereas Sonic Genetics and Australian Clinical Labs offer Harmony. As in other countries, other providers also perform NIPT.

## 3 PATENT LAW PRINCIPLES AND NIPT

Each patent application must be assessed on its own merits to determine validity in every jurisdiction in which it is filed. Once a patent is granted, the patent holder has the right to enforce it against alleged infringers. By asserting a patent, however, the patent holder provides the opportunity for other parties to argue that the patent is in fact invalid and should never have been granted. A number of arguments can be raised regarding validity, such as technical questions relating to the novelty, obviousness, and usefulness of the invention, and the adequacy of the disclosure relative to the scope of claims. However, it is the threshold question of whether the subject matter is inherently patent‐eligible that has attracted most attention in recent times, particularly in the context of DNA sequences and methods of their use.

Until recently, it was uncontroversial to observe that the law of the United States was more permissive towards the patentability of genetic subject matter than Europe, with Australia taking a middle road. However, a series of US cases has reversed this proposition, and as a result, US law now offers significantly less expansive patent eligibility than previously.^11^, ^12^, ^13^, ^14^ These decisions have created uncertainties because of the ambiguity of their applicability.^15^ In order to fully appreciate the nuances of these decisions, it is first important to clarify the distinction between product and method patents.

Product patents claim protection over physical entities or things—“compositions of matter.” Such claims have been controversial in genetics, with the primary concern being that broad product claims vest control of all uses of the particular products in the patent holder. Depending on how these patent rights are managed and enforced, follow‐on research and development can be stifled. In the context of DNA sequence claims, a patent could potentially block development and use of all diagnostic tests and therapeutics that entail production of all or part of the sequence.^16^, ^17^, ^18^, ^19^, ^20^

In the NIPT context, however, the direct implications of product patents are actually relatively small, as the vast majority of NIPT patents make claims on methods. Method patent claims can have significant impact on development and use of genetic diagnostic tests. Indeed, a study conducted by Huys et al found that method claims were more prone to block diagnostic uses than sequence claims.^21^

## 4 PATENT PRACTICE IN THE UNITED STATES

In the United States, patents on the *BRCA1* and *BRCA2* genes associated with inherited risk of breast and ovarian cancer included broad product claims on isolated and human made DNA sequences (respectively, gDNA and cDNA), as well as a variety of method claims. Ultimately, the Supreme Court was only required to consider the product claims, because lower courts invalidated the broad method claims.^22^ The Court held in the *Myriad* decision that “A naturally occurring DNA segment is a product of nature and not patent eligible merely because it has been isolated, but cDNA is patent eligible because it is not naturally occurring.”^13^ The Supreme Court did not explain, however, how to determine the change required to the DNA sequence (or other naturally occurring substance) to make it “markedly different” 23 from its naturally occurring counterpart, and hence, patent eligible. This question, and the broader implications of the *Myriad* decision for medical biotechnology, has been the subject of a great deal of criticism and commentary.^20^, ^24^

Patent eligibility for DNA‐related methods has been narrowed under US law by three key cases.^11^, ^12^, ^14^ The most relevant is *Mayo v Prometheus,* 25 which concerned claims to a method for titrating the dose of thiopurine drugs by measuring a specific metabolite. The Supreme Court invalidated the method claims, as merely applying a law of nature,^25^and held that a process which applies a law of nature will not be patentable unless that process has additional features beyond those already known in the field. *Mayo* was not a gene patent case, but it had important implications for DNA diagnostic method claims in patents such as those held by Myriad, the key diagnostic claims of which were invalidated by the lower courts. Following *Mayo* and other cases, the US Patent and Trademark Office (USPTO) issued a set of guidelines to assist examiners in determining patent eligibility.^26^ Examiners must now decide whether a claim is directed to a “judicial exception” (in life sciences, a law of nature or a natural phenomenon) or is “markedly different” from the exception (Step 2A). If the claim is directed towards one of the recognised exceptions, or is not markedly different from them, the examiner must determine whether there are elements in the claim, alone or in combination, that add “significantly more” than the judicial exception, going beyond well understood, routine activities in the relevant art (Step 2B). Therefore, following these decisions, it is substantially more difficult to patent a diagnostic method in the United States.

The limitations imposed by the *Mayo* line of reasoning are very important for NIPT patents, and were tested by the US Court of Appeals for Federal Circuit in *Ariosa v Sequenom*.^27^, ^28^ After a complicated route through the courts, the Federal Circuit invalidated the patent, finding that the relevant claims were directed to naturally occurring phenomena (Step 2A), and that the claims included no inventive concept sufficient to transform the claimed naturally occurring phenomena into a patent eligible application (Step 2B).^29^ The US Supreme Court declined to review the decision of the lower court,^30^ so the 540 patent remains invalid in the United States, which means that other players can offer their tests without fear of infringing this particular patent.

In December 2018, two patents held by Illumina were invalidated for lack of patent‐eligible subject matter.^31^ Illumina and Natera are involved in litigation over a patent for NIPT library preparation.^32^ Future cases may arise involving other NIPT patents.

## 5 PATENT PRACTICE IN EUROPE

In Europe,^33^ a patent must be for an “invention” in order to constitute patentable subject matter, and the European Patent Convention (EPC) specifies that a discovery is not an invention.^34^ However, a useful artefact or process that results from a discovery can constitute patentable subject matter, 35, 36 and it is the practical application of an idea or discovery which leads to patentability.^37^ The European Patent Office (EPO) Boards of Appeal and courts in most European countries have taken a relatively expansive approach to patentable subject matter, but have restrictively applied patent criteria for novelty, inventive step, and insufficient disclosure.^19^ In practice, this tends to mean that patents are scrutinised closely to determine whether they are truly new, non‐obvious, and that the disclosure of the invention is sufficient to allow others to perform it, rather than a blanket exclusion for whole classes of inventions as “natural” such as under current US practice.^38^

Under current interpretation in Europe, in contrast to the United States, isolating DNA confers sufficient distinction from its natural state for it to be patentable. Uncertainties and divergence in the law among European jurisdictions were directly addressed in the provisions of the Biotechnology Directive,^39^ notably Article 5, where a distinction is drawn between naturally occurring substances and the products which result from the human effort involved in isolating those substances from their natural environment.^40^ Where a DNA sequence is isolated from the human body by means of a technical process, the sequence (substance) *per se* becomes eligible for patent protection,^41^ even if it is identical to that which occurs in vivo.^42^ Given that these provisions codify that isolation is sufficient to confer the necessary technical character for patentability, it is unlikely that the US *Myriad* decision will be followed in Europe.

With regard to method claims, European cases have focused on whether there is a technical contribution. This technical contribution may be fairly minimal, but provided there is a technical contribution the invention will be patentable and not excluded subject matter “as such.”^43^, ^44^

In Europe, a patent granted by the EPO may be opposed in proceedings at the EPO, where a patent may be struck down in whole or in part. If that opposition is unsuccessful, the patent may also be challenged later in national courts. The European patent in the 540 family (EP 0994963) was opposed, but was upheld in opposition proceedings and on appeal to the Technical Board of Appeal of the EPO.^45^ Unlike in the United States, the proceedings did not address the question of subject matter eligibility. Instead, the patent was opposed on the grounds of lack of inventive step and insufficient disclosure. The Board of Appeal found that there was sufficient disclosure of the invention in the patent to enable a person in the same technical field to perform it, and that the claims in question were not obvious and therefore inventive enough to satisfy the patentability requirements.

The patent is now being contested in national proceedings in various European countries. A preliminary injunction was granted against molecular diagnostic company Amedes MVZ Trägergesellschaft and a related company performing an NIPT (based on Ariosa technology) in Germany.^46^ The UK 540 patent (Lo 1) and other patents arising from the work of Dennis Lo (Lo 2 and 3) and Stephen Quake (Quake patents) were contested by Premaitha Health, TDL Genetics, and Ariosa Diagnostics in proceedings before the UK High Court in 2017.^47^ An infringement action had been brought against these firms by Illumina, Sequenom, and others. In counterclaim, a plethora of legal issues were raised. The question of whether the subject matter of the 540 patent constituted a discovery as such, rather than an invention, was disposed of in a single paragraph.^48^ The patent was held not to be a discovery, because the claims are not directed to information about the natural world, but rather to a practical process, the detection method.^48^ Nevertheless, the court found that the Lo 1 patent was valid only in part, based on failure to satisfy some of the technical patent criteria, and significantly narrowed the scope of the patent Premaitha was held to infringe the Lo 1 patent with its IONA test, although not with its alternative proposed process (the Additional Alternative Proposed Process). In contrast, TDL and Ariosa's Harmony test was found not to infringe. The Lo 2 and Lo 3 patents were affirmed as valid, as were Quake patents (with permitted amendments), and the IONA test was held to infringe all, including Lo 1. In 2018, Illumina and Premaitha settled all litigation and Premaitha licensed Illumina's patent pool.^49^ However, like the United States, there are other NIPT cases in progress.

## 6 PATENT PRACTICE IN AUSTRALIA

Patent 727919 is Australia's member of the 540 patent family. In 2016, Sequenom filed an infringement suit against Ariosa Diagnostics, Sonic Healthcare and Clinical Laboratories in the Federal Court of Australia. After a trial in 2018, judgement is reserved.

Australia has a broad requirement that subject matter is a “manner of manufacture”, akin to the “composition of matter” requirement in US law. A 1959 decision of the Australian High Court in *National Research and Development Corporation v Commissioner of Patents* (*NRDC*)^50^ remains the main authority on interpreting the manner of manufacture requirement. Here, the claimed process was held to be patentable because it produced a product that was an “artificially created state of affairs” and “the significance of the product [was] economic.” Over the years since NRDC, these twin requirements have largely determined the manner of manufacture requirement.

In litigation relating to the Australian patent corresponding to those considered in the US *Myriad* case, the only claim that was challenged was a DNA sequence claim. The High Court of Australia unanimously invalidated that claim in the Myriad patent, and held that isolated DNA sequences are not patentable subject‐matter.^51^ The High Court noted that satisfaction of the manner of manufacture test requires that something is “made,” and that this “must be something brought about by human action.”^52^ The court held that the substance of the claims in the Myriad patent was the information embodied in the arrangements of nucleotides, and this information was not made by human action, but was instead discerned.^24^

While there have been some significant method‐related Australian cases, until recently, none considered the specific issues raised in the US case of *Mayo* about the patentability of methods applying a law of nature. Beach J of the Australian Federal Court considered methods of this nature in *Meat and Livestock Australia v Cargill* (*Cargill*).^53^ The patent in issue included a series of method claims for identifying bovine traits from nucleic acid samples using single nucleotide polymorphisms for managing, selecting, breeding, and cloning cattle. His Honour rejected arguments that the claims involved simply the practical application of a naturally occurring phenomenon to a particular use, and the patent was not invalidated on this basis.^54^ Beach J found that the test in *Mayo* was “too sweeping for [him] to work out whether [he was] acting consistently or inconsistently with its spirit” when determining what it takes to transform an unpatentable law of nature into a patent‐eligible application^55^

Although the *Cargill* decision delivered by Beach J is likely to be appealed, it is notable that he is the judge who has also been allocated the *Sequenom v Ariosa* trial. That litigation will present Australian courts with an opportunity to consider the patent‐eligibility of method claims in the context of NIPT. Even so, it is likely to take several years before there is a definitive ruling as to the patentability of methods of diagnosis in Australia, assuming that the parties pursue court proceedings and appeals with the same passion as they have done in the United States.

## 7 IMPLICATIONS FOR THE FIELD

As is evident, there are many patents in the field of NIPT. The 540 patent has already been invalidated in the United States, with the corresponding patents the subject of ongoing litigation in Europe and Australia. In any case, the patent expired in 2017, although the damages payable for infringement during its life are considerable, making continuing litigation worthwhile. Moreover, there are also other patents relevant to NIPT held by a number of parties that are in litigation, and others still are not yet being litigated. Some of these form part of the patent cross‐licensing between Sequenom and Illumina, and others are held by other parties offering commercial testing. Many of these patents are likely to be valid and enforceable, at least in part, and in some jurisdictions.

The validity of patents varies across different jurisdictions, with implications for the global NIPT market. At present, there is the distinct possibility that patents that are valid in Europe and Australia may be invalid in the United States. A valid patent gives the rights‐holder the ability to limit the actions of competitors or to require licenses and royalty payments in the jurisdictions where that patent is valid. A patent position therefore confers a commercial advantage, but it remains to be seen how variation in patent portfolios internationally might influence competitive strengths in the global NIPT market. Indeed, some companies appear to be targeting markets where there is greater freedom to operate in the absence of patents held by their competitors (eg, in the Middle East or in parts of Asia), with the arguable collateral benefit of increased access in markets that have traditionally been lesser served by biomedical innovation. At present, there is uncertainty while court decisions remain outstanding. As litigation is resolved, either through judgments or through settlements, the relationships between patent holders and users of patented technology will become more certain. However, commercial uncertainty will operate as a background to the delivery of tests for some time.

The many patents held by multiple parties make it likely that in the long run, numerous providers will remain in the market. The extensive patent portfolio and cross‐licensing agreement between Illumina and Sequenom, which includes the 540 patent, will likely afford them a strong position in the market, and the ability to demand licensing from other NIPT providers (or even prevent them operating in some jurisdictions). Although other patents are also significant, they are narrower in scope, and therefore are unlikely to enable one party to operate to the exclusion of all others. Thus, several parties will likely continue to offer testing, with cross‐licensing or modification of testing methods to avoid infringement as necessary.

In addition to the commercial providers offering NIPT, many public sector providers also perform NIPT, either as partners with the commercial providers or by developing their own NIPT. Increasingly, commercial entities are entering new markets where they have not previously had a large presence, including, for example, some European countries where genetic testing has been almost exclusively through public sector laboratories. The nature of NIPT technology, coupled with patents, means that commercial parties have been able to bargain for samples to be sent to their laboratories for testing or for the licensing of their “black box” technology transfer into public sector laboratories.

The translation of NIPT technology into clinical application has been very rapid. Pregnant women may access NIPT on a private, pay for service basis, or through insurance coverage. Increasingly, public healthcare systems, especially in Europe, provide access to NIPT testing for certain patient groups,^56^, ^57^ with the likelihood that other countries will roll out public sector programmes in the next few years.^58^, ^59^ However, access to NIPT is limited on the basis of cost in many countries. The contribution of patents to the cost of testing is a complex economic question, and there is no direct or linear relationship. However, it is inevitable that the high costs of litigation and patent enforcement will be passed on to those who pay for tests. At the same time, the competition between numerous commercial parties offering testing will serve to keep commercial prices down. While it is not possible to offer definitive answers about the impact of patents on patient access to testing, as that is dependent on factors including how private and public payors cover these tests, it will continue to be important to monitor options of technologies and tests available to public healthcare systems, as the patent issues are resolved through judicial decisions and settlements.

## 8 CONCLUSION

The patent situation in NIPT remains in flux. Uncertainty in relation to the enforceability and scope of patents will persist for the foreseeable future. Patents are unlikely to result in a monopoly for a single provider, however, given the cross‐licensing of crucial patents, jurisdictional differences in patent eligibility, the plurality of providers and testing technologies, and the diverse public and private sector entities involved in NIPT.

## FUNDING STATEMENT

NH is supported by the UK Economic and Social Research Council (grant code ES/K009575/1). DN is supported by funding from the Australian Research Council, DP180101262. RC‐D and SC were supported by NIH grant R01 HG007074, “Intellectual Property and Access to Noninvasive Prenatal Testing.”

## CONFLICT OF INTEREST STATEMENT

The authors have stated explicitly that there are no conflicts of interest in connection with this article.

## DATA AVAILABILITY STATEMENT

Data sharing is not applicable to this article as no new data were created or analyzed in this study.
